# Single-Cell Sequencing: Current Applications in Precision Onco-Genomics and Cancer Therapeutics

**DOI:** 10.3390/cancers14030657

**Published:** 2022-01-28

**Authors:** Lisa Maria Mustachio, Jason Roszik

**Affiliations:** 1 Department of Epigenetics and Molecular Carcinogenesis, The University of Texas MD Anderson Cancer Center, Houston, TX 77030, USA; 2 Center for Cancer Epigenetics, The University of Texas MD Anderson Cancer Center, Houston, TX 77030, USA; 3 Department of Genomic Medicine, Division of Cancer Medicine, The University of Texas MD Anderson Cancer Center, Houston, TX 77030, USA; 4 Department of Melanoma Medical Oncology, Division of Cancer Medicine, The University of Texas MD Anderson Cancer Center, Houston, TX 77030, USA

**Keywords:** single-cell sequencing, large integrated databases, immunotherapy, personalized medicine, cancer

## Abstract

**Simple Summary:**

Single-cell sequencing technologies are growing, advancing, and supporting new opportunities to better understand cancer. A variety of technologies are available that analyze the human transcriptome, genome, epigenome, and proteome, enabling integrated datasets. As a result, these integrated datasets contribute to new mechanistic insights and areas with therapeutic potential. This review summarizes the various single-cell sequencing techniques and provides examples of recent high-impact findings from the utilization of these technologies. Additionally, the translational relevance of these technologies and their use in clinical trials is described, along with the future potential for novel findings using these innovative methods.

**Abstract:**

Single-cell sequencing encompasses a variety of technologies that evaluate cells at the genomic, transcriptomic, epigenomic, and proteomic levels. Each of these levels can be split into additional techniques that enable specific and optimized sequencing for a specialized purpose. At the transcriptomic level, single-cell sequencing has been used to understand immune-malignant cell networks, as well as differences between primary versus metastatic tumors. At the genomic and epigenomic levels, single-cell sequencing technology has been used to study genetic mutations involved in tumor evolution or the reprogramming of regulatory elements present in metastasized disease, respectively. Lastly, at the proteomic level, single-cell sequencing has been used to identify biomarkers important for predicting patient prognosis, as well as biomarkers essential for evaluating optimal treatment strategies. Integrated databases and atlases, as a result of large sequencing experiments, provide a vast array of information that can be applied to various studies and accessed by researchers to further answer scientific questions. This review summarizes recent, high-impact literature covering these aspects, as well as single-cell sequencing in the translational setting. Specifically, we review the potential that single-cell sequencing has in the clinic and its implementation in current clinical studies.

## 1. Single-Cell Sequencing Technologies and Integrated Databases

Single-cell sequencing evaluates heterogeneity in cellular populations at the transcriptomic, genomic, epigenomic, and proteomic levels [1,2]. This methodology is frequently used to understand changes that occur in disease states and is especially helpful for analyzing tumors that exhibit various morphological and phenotypic profiles. Over the years, this technology has rapidly advanced to generate multiple single-cell sequencing techniques, all of which are used for specialized and unique purposes. There are a variety of current technologies that help understand individual profiling (i.e., profiling at the transcriptomic level) or multiomics profiling (i.e., profiling at the transcriptomic and genomic levels) of single cells [3] that we briefly summarize in this review.

**Transcriptomic Analysis:** Unlike RNA sequencing (RNA-seq) that measures transcripts in a group of different cell types, single-cell RNA sequencing (scRNA-seq) evaluates the transcriptomic status of specific populations of single cells [3,4]. Microdroplet and microwell protocols allow for the simultaneous barcoding and handling of thousands of single cells [3]. Technologies, such as Smart-seq, Smart-seq2, Quartz-Seq and CEL-seq, measure mRNAs that are isolated from a single cell [1,3,5]. These various mRNA sequencing methods achieve different purposes. For example, Smart-seq technologies measure full-length transcripts. Quartz-Seq analyzes the 3′ end of transcripts and CEL-seq barcodes and pools samples before mRNA linear amplification [1]. A recent study transcriptionally profiled 371,223 cells from colorectal cancer and adjacent normal tissues of 28 mismatch repair-proficient and 34 mismatch repair-deficient tumors [6]. Interestingly, this work showed that T cells are organized in structured cell neighborhoods within a tumor. Overall, the authors provide datasets containing cellular states, gene networks, and tumor transformations across a large number of individuals with colorectal cancer. Hubs of interacting malignant and immune cells were identified to understand spatially organized immune-malignant cell networks. Studies such as this demonstrate the power of this technology for analysis of interacting cellular programs. Transcriptomic analysis is also being interrogated to understand metastatic cancers and how primary tissues compare with micrometastases. Using single-cell RNA sequencing methods and looking at patient-derived-xenograft models of breast cancer, one group uncovered that both primary tumors and micrometastases showed transcriptional heterogeneity [7]. However, it was found that tissues showing micrometastases displayed a distinct profile that predicted poor prognosis. Specifically, this distinct profile in micrometastases contained mitochondrial oxidative phosphorylation as a top pathway upregulated in these tissues. This indicates that targeting pathways involving mitochondrial oxidative phosphorylation may attenuate metastases observed in breast cancer patients. Lastly, transcriptomic analysis is being applied to tumor evolution. A robust study published in *Cancer Cell* analyzed the transcriptome of mouse lung tumors using scRNA-seq. The transcriptome was analyzed starting from the pre-neoplastic state all the way to advanced states, such as adenocarcinoma [8]. The authors revealed that diversity in transcriptomics increases as the tumor progresses into advanced stages. They also uncovered that a high-plasticity cell state (HPCS) is adopted during the tumorigenesis process, and this HPCS displays a high capacity for differentiation along with a high proliferation rate. A HPCS was also found to increase chemoresistance in mice, as well as contribute to their poor survival. Transcriptomics and multiplexed ion beam imaging combined with scRNA-seq were also used to study human cutaneous squamous cell carcinoma (cSCC) and normal skin tissues [9]. Tumor and stromal cell subpopulations, spatial niches where interactions occur and communicating gene networks were all identified. In addition, using a combination of single-cell and spatial data allowed for the mapping of ligand-receptor networks to specific cell types in the context of a tumor. For example, tumor keratinocyte ligands were used to predict modulation of tumor microenvironment-specific cell-type signatures. Similarly, scRNA-seq has been used to understand the molecular and cellular dynamics in metastatic lung cancer to uncover new diagnostic and therapeutic targets [10]. Cancer cell subtypes were identified from 208,506 cells in normal tissues all the way to tissues showing signs of early metastatic disease. Both immune and stromal changes were observed throughout this trajectory. This helps set the stage for how the microenvironment becomes both immunosuppressive and pro-tumoral as a tumor begins to advance.

**Genomic Analysis**: Single-cell whole-genome sequencing (scWGS) methods are used to evaluate germline or somatic mutations [11]. More specifically, the multiple displacement amplification (MDA), multiple annealing and loop-based amplification cycles (MALBAC), degenerate oligonucleotide-primed PCR (DOP-PCR) and PicoPLEX techniques are all used to uniformly amplify genomic DNA in individual cells [1,3]. Approaches that combine transcriptome and genome sequencing in single-cell multiomics analyses have been generated, including single-cell triple omics sequencing (scTrio-seq), genome and transcriptome sequencing (G&T-seq), gDNA-mRNA sequencing (DR-seq), simultaneous isolation of genomic DNA and total RNA (SIDR) and TARGET-seq [1,3,12]. By combining transcriptome and genome approaches, a deeper and more comprehensive analysis of cellular activities can be performed to better understand normal versus pathological states [1,12]. In addition, key data that may not be apparent in transcriptome studies, may be evident in genomic approaches, or vice versa. scWGS methods are being used to understand how mutations in B lymphocytes are linked to aging and cancer [13]. Interestingly, mutations in human B lymphocytes were found to significantly increase with age, reflecting genetic signatures closely related to B cell cancers. scTrio-seq has been used to understand colon cancer lineages to study mutations, the transcriptome and the methylome in colon cancer primary tumors and metastatic tumors from patients [14]. This work found that the DNA demethylation degrees in cancer cells correlated with the density of the heterochromatin-associated nuclear element 1. During tumorigenesis and progression, heterochromatin regions exhibit aberrant DNA demethylation. The study presented here reveals new discoveries for the understanding of tumor evolution and the link between DNA methylation and genetic lineages.

**Epigenomic Analysis**: The cell lineage and differentiation status of individual cells can be analyzed using single-cell sequencing epigenomic technologies investigating DNA methylation and chromatin states [1]. Single-cell bisulfite sequencing (scBS-seq), single-cell reduced representation bisulfite sequencing (scRRBS), single-cell whole-genome bisulfite sequencing (scWGBS), single-nucleus methylcytosine sequencing (snmC-seq) and single-cell combinatorial indexing for methylation (sci-MET) are all used to measure DNA methylation profiling [15]. To measure histone protein modifications, droplet-based chromatin immunoprecipitation (Drop-ChIP) captures a single cell in a droplet containing a cell-specific barcode to generate chromatin fragments that can then be analyzed in ChIP-seq [1,3]. To study open chromatin, various techniques, such as single-cell DNase sequencing (scDNase-seq), single-cell combinatorial indexing assays for transposase-accessible chromatin with sequencing (sci-ATAC-seq), single-cell assays for transposase-accessible chromatin using sequencing (scATAC-seq), nucleosome occupancy and methylation sequencing (NOMe-seq) and single-cell micrococcal nuclease sequencing (scMNase-seq) are used [16]. There are several methods combining the analysis of chromatin, along with the transcriptome, including single-cell combinatorial indexing chromatin accessibility and mRNA (sci-CAR), single-nucleus chromatin accessibility and mRNA expression sequencing (SNARE-seq) and single-cell nucleosome methylation and transcription sequencing (scNMT-seq) [1,3]. Epigenomic datasets were produced with the use of bisulfite and ChIP-seq in normal prostate tissues, as well as localized versus metastasized prostate cancers to find reprogrammed regulatory elements present in metastasized disease [17]. In addition, the presence of metastasis-specific enhancers can be uncovered using these epigenomic datasets [17]. Altogether, these datasets contribute to the understanding of the epigenetics behind prostate cancer oncogenesis. Epigenetic studies such as this can be applied to other cancers as well.

**Proteomic Analysis**: Single-cell proteome sequencing is more difficult to accomplish. One reason for this is that proteins cannot be amplified like mRNA or DNA [1,18]. However, there are methods that measure the transcriptome and proteome of a single cell. These methods include the proximity ligation assay for RNA (PLAYR), proximity extension assay/specific RNA target amplification (PEA/STA), cellular indexing of transcriptomes and epitopes by sequencing (CITE-seq) and finally, the RNA expression and protein sequencing assay (REAP-seq) [1]. One group generated single cell transcriptome to protein prediction with a deep neural network (cTP-net) to impute surface protein abundances for scRNA-seq data [19]. Results from this study indicated that REAP-seq and CITE-seq data can be used to predict surface protein abundances for new scRNA-seq datasets. CyToF is a mass cytometry method that analyzes multiple proteins tagged with labels. It has previously been alternatively used or combined with scRNA-seq in cancer immunity studies [3,20]. For example, single-cell mass cytometry (CyToF) is commonly used to classify cell surface proteins of immune cells into specific cell classifications. This is critical for studies investigating immune cell compositions in the tumor microenvironment. Although not at the single-cell level, mass spectrometry approaches are commonly used for large proteomic analyses and may provide value in studies where single-cell proteome sequencing is not feasible. Proteomic analysis using the mass spectrometry approach parallel reaction monitoring (PRM) of extracellular vesicles and particles (EVPs) was performed in ~400 human samples to reveal that EVP proteins can be used as biomarkers for cancer detection and for determining cancer type [21]. The Cancer Cell Line Encyclopedia (CCLE) has recently been expanded through quantitative profiling using mass spectrometry of proteins across 375 cell lines to reveal additional information that has not yet been uncovered using RNA or DNA technologies that aid in cancer research [22]. New correlations not available by interrogating RNA methods were uncovered. Specific protein complexes were found to be associated with translation and mutations.

**Large Integrated Databases and Atlas Projects**: With the development of new single-cell sequencing techniques and the generation of large data sets, there has been collaboration and integration to produce publicly available databases. The Human Cell Atlas (HCA) was designed to generate reference maps of all human cells by providing open single-cell genomics data sharing [23]. The many studies included in the HCA are available at https://data.humancellatlas.org/explore/projects, accessed on 14 January 2022. Single cell sequencing data compiled by the HCA reflects information for many diseases, including cancer. For example, a recent cervical cancer study included in the HCA project compared tumor-derived endothelial cells to normal endothelial cells. With the use of scRNA-seq, this study identified that there was a higher expression of metabolism-related genes in tumor-derived endothelial cells [24]. Overall, intra-tumoral heterogeneity and transcriptional activities of endothelial cells in cervical cancer were nicely identified. Another study included in the HCA project characterized tumor-infiltrating immune cells using single cell RNA-seq. This study successfully profiled over 40,000 immune cells from eight breast cancer carcinomas, and matched normal breast tissues, blood and lymph nodes [25]. Increased heterogeneity of intratumoral cells of both lymphoid and myeloid cell lineages were observed. This resulted in the conclusion that continuous phenotypic expansions specific to the tumor microenvironment are apparent relative to normal tissues, highlighting the importance of the characterization of tumor-infiltrating immune cells.

An extensive cell atlas of the human lung was created using droplet- and plate-based single cell RNA sequencing of 75,000 human cells across all lung tissue compartments, along with a multi-pronged cell annotation approach [26]. This lung cell atlas will aid in identification of functions and interactions achieved through development and disease states. More specifically, it revealed the presence of a high level of plasticity of cell-types and specific gene expression profiles during the evolution of the lung. Similarly, a large-scale, single-cell atlas for breast cancer ecosystems was generated to identify new precision medicine approaches [27], while another study used scRNA-seq data to identify unique breast epithelial clusters from healthy breast tissues [28]. Interestingly, certain genes were found to be co-expressed along with the estrogen receptor (ER) in a large number of breast cancer tissue samples, indicating a clinically relevant subclassification of ER positive breast cancers. Lastly, a single-cell atlas for osteosarcoma has been generated to explore intratumor heterogeneity and provide new therapeutic strategies for this cancer-type [29].

Multiple other single-cell atlases have also been created to study the composition of normal tissues and to better understand the physiology of healthy states versus disease states. One study combined human liver scRNA-seq data to characterize heterogeneity between the different datasets and define dominant phenotypes across immune cell populations [30]. This work was performed in the healthy liver to better understand immune-related diseases that occur in this tissue. Another study used four scRNA-seq datasets to gain insight into normal liver architecture and gene expression and to generate a portal [31]. This portal contains updated information and allows for the compilation of a comprehensive array of data available to any scientist investigating liver diseases. Compiled datasets and portals such as this allow for the analysis and comparison of specific genes across the different liver datasets. Some other examples include a single cell transcriptomic atlas analyzing different human cardiac arteries to identify cell populations associated with vascular physiology [32]. A kidney single-cell atlas identified myeloid heterogeneity in the progression and regression of kidney disease [33]. A single-cell transcriptome atlas of the adult human retina identified multiple cell populations to better understand retinal biology and disease [34]. Furthermore, a single-cell atlas was generated to study the differences between mouse and human prostate tissues to investigate heterogeneity and conservation of epithelial progenitors [35].

Not only has single-cell transcriptome profiling been used to study a single organ at a time, it has also been implemented to identify the characteristics of 15 different human organs [36]. This helps better understand the mechanisms behind disease in multiple tissues. The Single Cell Type Atlas of the Human Protein Atlas aims to map all human proteins by integrating antibody-based imaging and RNA-seq technologies [37]. By compiling multiple tissues into one platform, an immense amount of data will be available to identify interesting associations and relationships. There is a surge of novel work generating these atlases that will provide a rich array of information to understand multiple cancer-types. These atlases will also provide knowledge for cancer-related mechanisms and new ways for targeted therapy. As discussed, they will also provide information on normal tissue physiology to better understand how disease states form in the first place. The number of new publications presenting single-cell atlases over the last couple of years is striking compared to the number published over 5 years ago. Table 1 summarizes examples of the different single-cell atlases and large integrated data bases created over the last few years. This table includes the data sets that are discussed in this review.

## 2. Translational Relevance of Single-Cell Sequencing Technologies

**Figure 1:**
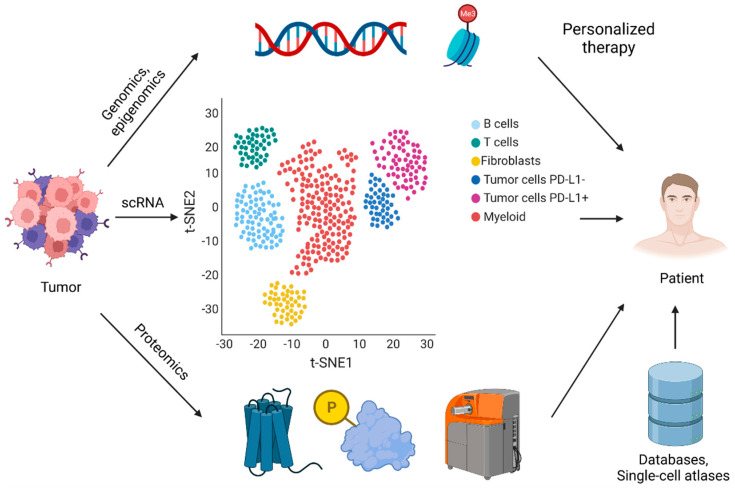
An illustration of using single-cell technologies and databases for personalized cancer therapy.

As previously mentioned, single-cell sequencing data can help predict alterations present at the transcriptomic, genomic, epigenomic, and proteomic levels in healthy versus malignant cells. There are several reports using single-cell analysis to analyze the tumor microenvironment [38] and to measure the presence of certain cell types before and after treatment with specific therapeutics. One example is a study performed on a treatment-refractory bladder cancer patient. In this study, scRNA-seq was used to understand the tumor microenvironment [39]. Similarly, single-cell analyses were performed in renal cell carcinomas compared to benign kidney tissues. This work provided insight into the biology behind how renal cell carcinoma develops and how it responds to therapy [40]. Furthermore, single-cell profiling is being ran to uncover molecular checkpoint or activation targets within tumors and to identify how a patient responds to targeting a certain protein or pathway. For example, genome wide analyses of DNA have been pursued to identify mutations that can tailor the way one is treated [41]. These strategies are critical in cases where patients are not responsive to standard treatments. Situations like this frequently occur as a result of the complexity and heterogeneity of diseased tissues between individuals. As a result, there appears to be a bright future for the use of scRNA-seq methods for personalized therapies (illustrated by Figure 1).

Single-cell sequencing has also been used to study circulating tumor cells. Specific modifications of single-cell sequencing technologies have been produced to better handle these studies. A new technology that is termed Hydro-seq has been optimized to surpass issues of blood contamination frequently observed when using scRNA-seq. Hydro-seq can be used when studying circulating tumor cells and allows for high throughput analysis of these cells [42]. As shown in the example of breast cancer, Hydro-seq successfully enabled the high capture and purification of circulating tumor cells. More specifically, this study identified targets in breast cancer and tracked cell markers that can monitor metastasis as well as response to treatment [42]. The use of single-cell sequencing can also help identify new molecular signatures that can be used to analyze the response a patient has to a particular treatment. A study performed in myeloma identified the most significantly altered pathways during progression that can be used to determine prognosis and treatment stratification [43]. In another study, results from scRNA-seq revealed an upregulation of programmed death-ligand 1 (PDL1) [39]. As a result, the patient included in the study was treated with a PDL1 inhibitor. After treatment, a favorable response was observed. Single-cell technology has also been used to identify the presence of immunotherapy persister cells after PDL1 blockade, a population that may be targeted using combinational strategies [44].

Combinational strategies are often needed, and single-cell analysis may help identify potential combinational therapies. Identifying new combinations helps tremendously with immunotherapy strategies where most patients do not respond to monotherapy as a first-line treatment [45]. In a recent study, single-cell RNA sequencing was performed to show that a combinational strategy using both an RAS inhibitor and IR820 nanocapsule-augmented sonodynamic therapy suppressed hepatocellular carcinoma and modulated immunity through differentially expressed genes [46]. In a similar study, single-cell sequencing was performed on CD45^+^ tumor-infiltrating lymphocytes (untreated controls or PDL1 and AB680 CD73 inhibitor treated) to find that CD73 inhibition is distinct from PDL1 inhibition [47]. This work also observed that inhibition of CD73 along with PDL1 may produce a synergistic effect in colorectal cancer. Single-cell sequencing has recently also been used to describe changes in tumors after treatment. This technology is also utilized to identify how these changes are associated with drug tolerance as well as inhibition of tolerant cell populations along with combination strategies [48]. A single-cell study performed in breast cancer tissues showed that there are pre-existing genotypes resistant to chemotherapy. These genotypes have the ability to further adapt after being exposed to chemotherapeutics. This contrasts with transcriptional profiles that can adapt in response to chemotherapy [49]. There is great promise that scRNA-seq studies will provide information for every cell type in a tumor where alterations may be associated with patient demographics, diagnostics, therapeutics, and prognostic factors. It is possible that in the near future, scRNA-seq will be used clinically to develop a personalized medicine regimen for the treatment of each individual patient. Tumors will be analyzed based on cell composition, along with specific proteins being expressed that can be targeted, and this may ultimately achieve a response that is optimal for each patient [41].

There are currently a handful of clinical trials being conducted world-wide that are implementing single-cell sequencing technology. In one clinical trial (Clinicaltrials.gov, accessed on 14 January 2022; Identifier NCT04927611), single-cell sequencing technology is being used to study the molecular features, tumor heterogeneity and cell subtypes of neuroendocrine neoplasms (NENs). Another trial (NCT04162691) aims to understand how genetics play a role in thymoma by understanding intratumoral heterogeneity using scRNA-seq. Lung cancer patients with bone metastases are being studied to identify a risk prediction model of bone-related events that can be based on single-cell sequencing (NCT04568291). Another ongoing trial is evaluating the proteomic and phenotypic signatures of prostate needle-core biopsies to better understand the malignant progression of prostate cancer (NCT02313623). Immune cell composition of bronchoalveolar lavage fluid from cancer patients diagnosed with cancer-therapy induced pneumonitis is being evaluated in the NCT04807127 trial to identify diagnostic biomarkers and therapeutic targets. A single-cell approach is being studied in NCT04807114 to identify biomarkers of efficacy and toxicity for immune checkpoint inhibitors in non-small cell lung cancer. Single-cell RNA sequencing is being used to explore the heterogeneity, identify tumor specific markers and explore the tumor microenvironment composition of lymphoma (NCT04434833). Gene expression at the single cell level is being analyzed in nasal and bronchial samples to understand the immune environment and complex interactions present in the respiratory tract (NCT04204291). Other trials are investigating the heterogeneity of dendritic cells in the colon and non-small cell lung cancer tumor microenvironment (NCT04789252) or the immune microenvironment in non-Hodgkin’s lymphoma patients (NCT04696692). Table 2 summarizes selected examples of the ongoing clinical trials adopting various single-cell sequencing technologies that are related to cancer therapy. There are other trials being performed using single-cell sequencing technology for the study of other diseases. These diseases include COVID-19 and inflammatory illnesses, such as psoriatic arthritis (Clinicaltrials.gov NCT04261010).

## 3. Conclusions

Despite the success and recent expansion of single-cell technology that allows us to seek answers to many previously unknown questions, the field of single-cell data science still faces challenges. These range from generating the best data possible to analyze, and consistency and integration with other datasets uploaded to databases. At the data generation level, mapping cells and states at specific resolution levels may pose a major challenge. Specific approaches, such as hierarchical stochastic neighbor embedding (HSNE), manifold learning and metric learning are being implemented to overcome this issue [50]. With an increase in resolution, there may be a decrease in the stability of supporting signals, which can lead to uncertainties regarding the data. Improved analysis tools are needed to ensure strong data quality. There is also an emphasis on defining flexible statistical frameworks to uncover complex gene expression patterns. Current methods can be improved through integration with approaches that acknowledge confounding variables and complex batch effects [50]. Also, better methods are needed to allow for the integration of single-cell data across experiments and measurement types. Lastly, we need to promote the collaboration of research communities to support the standardization of single-cell genomics data uploaded to consortiums and used by the research community [23]. As technology improves and more optimization studies are performed for specific purposes, modifications of current single-cell sequencing techniques will occur as shown in the case of Hydro-seq in the analysis of circulating tumor cells [42]. Another layer of challenges includes the difficulty in translating findings from sequencing studies into the clinic. Technical issues, such as variations in sample preparation and handling, must be addressed before streamlining and using these methods in a clinical setting world-wide [41]. It is also important that these methods are utilized in testing multiple, large enough cohorts to ensure reliable and accurate results [41]. After layers of validation, these methods can be routinely used in the clinic. It will be interesting to see what another decade of using this technology brings and how it can continue to improve the field of cancer therapy, as well as fields focused on other diseases, as these technologies help pave the way for the future of science and medicine.

## Figures and Tables

**Table 1:** A compilation of the summarized single-cell atlases and databases.

Site/Disease	Species	References
Cervical cancer	Human	[24]
Breast cancer	Human	[25,27,28]
Lung	Human	[26]
Osteosarcoma	Human	[29]
Liver	Human	[30,31]
Cardiac arteries	Human	[32]
Kidney	Human	[33]
Retina	Human	[34]
Prostate	Human, Mouse	[35]
15 organs	Human	[36]
13 tissues	Human	[37]

**Table 2:** Ongoing cancer-related clinical trials utilizing single-cell technologies.

Clinical Trial ID	Trial Name	Site/Disease
NCT04927611	Single-cell Sequencing and Establishment of Models in Neuroendocrine Neoplasm	Brain, neuroendocrine neoplasm
NCT04162691	Single Cell Sequencing Analysis of Thymoma	Thymus, thymoma
NCT04568291	CTC in Lung Cancer Patients With Bone Metastases	Lung cancer, bone metastasis
NCT02313623	MR-US Image Fusion Targeted Biopsy for Single-cell Prostate Cancer Research	Prostate cancer
NCT04807127	A Single-cell Approach to Identify Biomarkers of Pulmonary Toxicity for Immune Checkpoint Blockade	Lung, pneumonitis
NCT04807114	A single-cell Approach to Identify Biomarkers of Efficacy and Toxicity for ICI in NSCLC	Lung, non-small cell lung cancer
NCT04434833	A Single-cell Transcriptome Study in Patients with Non-Hodgkin’s Lymphoma	Lymph nodes, Non-Hodgkin’s Lymphoma
NCT04204291	Project A4sc- An Atlas of Airways at a Single Cell Level (A4sc)	Lung, chronic respiratory diseases
NCT04789252	Heterogeneity of Dendritic Cells in Colon and Non-small Cell Lung Cancer (TUM-DC)	Colon and Non-small cell lung cancers
NCT04696692	Single-cell Map of Immune and Lymphoma Cells in B-cell Non-Hodgkin’s Lymphoma (SIMILY)	B-cell Non-Hodgkin’s Lymphoma
NCT04261010	TNF and IL23 Blocking Agents Gene Expression Ratios in the Psoriatic Arthritis Synovium_(TIGERS) Study (TIGERS)	Psoriatic arthritis
